# *Bradyrhizobium septentrionale* sp. nov. (sv. septentrionale) and *Bradyrhizobium quebecense* sp. nov. (sv. septentrionale) associated with legumes native to Canada possess rearranged symbiosis genes and numerous insertion sequences

**DOI:** 10.1099/ijsem.0.004831

**Published:** 2021-06-09

**Authors:** Eden S. P. Bromfield, Sylvie Cloutier

**Affiliations:** ^1^​ Ottawa Research and Development Centre, Agriculture and Agri-Food Canada, 960 Carling Avenue, Ottawa, Ontario K1A OC6, Canada

**Keywords:** *Bradyrhizobium septentrionale *(sv. septentrionale), *Bradyrhizobium quebecense *(sv. septentrionale), insertion sequences, symbiosis genes, genetic rearrangements, Canada

## Abstract

Six bacterial strains isolated from root nodules of soybean plants that had been inoculated with root-zone soil of legumes native to Canada were previously characterized and 1) placed in two novel lineages within the genus *Bradyrhizobium* and 2) assigned to symbiovar septentrionale. Here we verified the taxonomic status of these strains using genomic and phenotypic analyses. Phylogenetic analyses of five protein encoding partial gene sequences as well as 52 full length ribosome protein subunit gene sequences confirmed placement of the novel strains in two highly supported lineages distinct from named *Bradyrhizobium* species. The highest average nucleotide identity values of strains representing these two lineages relative to type strains of closest relatives were 90.7 and 92.3% which is well below the threshold value for bacterial species circumscription. The genomes of representative strains 1S1^T^, 162S2 and 66S1MB^T^ have sizes of 10598256, 10733150 and 9032145 bp with DNA G+C contents of 63.5, 63.4 and 63.8 mol%, respectively. These strains possess between one and three plasmids based on copy number of plasmid replication and segregation (*repABC*) genes. Novel strains also possess numerous insertion sequences, and, relative to reference strain *Bradyrhizobium diazoefficiens* USDA110^T^, exhibit inversion and fragmentation of nodulation (*nod*) and nitrogen-fixation (*nif*) gene clusters. Phylogenetic analyses of *nodC* and *nifH* gene sequences confirmed placement of novel strains in a distinct lineage corresponding to symbiovar septentrionale. Data for morphological, physiological and symbiotic characteristics complement the sequence-based results. The data presented here support the description of two new species for which the names *Bradyrhizobium septentrionale* sp. nov. (sv. septentrionale) and *Bradyrhizobium quebecense* sp. nov. (sv. septentrionale) are proposed, with 1S1^T^ (=LMG 29930^T^=HAMBI 3676^T^) and 66S1MB^T^ (=LMG 31547^T^=HAMBI 3720^T^) as type strains, respectively.

The genus *Bradyrhizobium* is a large and diverse group of species and includes members that possess accessory genes for nitrogen fixation and symbiotic interaction with legume plants such as soybeans [1, 2].

In a previous study [3] an assessment was made of the diversity and evolutionary relationships of soybean nodulating bacteria associated with legumes native to eastern Canada. Two soybean cultivars were used to selectively trap bacteria from root zone soils of different native legume species and more than 800 bacterial isolates were obtained from root nodules of soybean plants. These bacterial isolates were characterized by multiple locus sequence analysis (MLSA) of five protein encoding core genes and multiple novel lineages of *Bradyrhizobium* were identified. Bacterial strains representing several of these lineages were assigned to a symbiotic ecotype or symbiovar (sv.) that we named septentrionale (formerly septentrionalis) based on the finding that they possessed unique nodulation (*nod*C) gene sequences and distinctive symbiotic characteristics.

During the course of the present work we detected high numbers of insertion sequences (small transposable elements, usually less than 3 kb in size) in the genomes of novel strains, a situation similar to that for strains of *Bradyrhizobium* sp. that possess highly reiterated sequences and were isolated from root nodules of soybeans grown in Japan [4]. Insertion sequences are important because they play a significant role in bacterial evolution by inducing mutations, deletions and rearrangements within the host genome [5].

In the work described here we used phylogenetic, genomic and phenotypic analyses to further characterize strains representing two novel *Bradyrhizobium* lineages. Based on the results the novel species *Bradyrhizobium septentrionale* sp. nov. (sv. septentrionale) and *Bradyrhizobium quebecense* sp. nov. (sv. septentrionale) are proposed.

## Habitat and isolation

Novel strains were isolated from root nodules of soybean plants that had been inoculated with suspensions of root-zone soil from the following native legumes growing at woodland sites in the province of Québec, Canada as previously described [3]: *Amphicarpaea bracteata* (hog-peanut) (site S1, Gatineau, and, site S4, Donnacona); *Desmodium canadense* (showy tick-trefoil) (site S2, Gatineau); and *Apios americana* (potato bean) (site S5, Québec). Plants of the native legume species at these sites were vigorous, showed no signs of nitrogen deficiency and the roots were extensively nodulated by resident soil bacteria.

Novel strains 1S1^T^, 162S2, 75S4 and 28S5 (*Bradyrhizobium septentrionale* sp. nov.) were from root-zone soils of the native legumes at sites S1, S2, S4, and S5, respectively whereas strains 66S1MB^T^ and 12S5 (*Bradyrhizobium quebecense* sp. nov.) were from root zone soils of the native legumes at sites S1 and site S5, respectively.

Novel strains 1S1^T^, 162S2 and 66S1MB^T^ were deposited in the BCCM/LMG Bacteria Collection, University of Ghent, Belgium (LMG collection nos. 29930^T^, 31550 and 31547^T^, respectively) and in the HAMBI Microbial Culture Collection, University of Helsinki, Finland (HAMBI collection nos. 3676^T^, 3724 and 3720^T^, respectively).

## Phylogenetic analyses of partial gene sequences

Sequences of 16S rRNA, *atpD*, *glnII*, *gyrB*, *recA* and *rpoB* core genes were used for phylogenetic analyses. Nucleotide sequence accession numbers are shown in Table S1. Alignment of 16S rRNA gene sequences was carried out using fast, secondary-structure aware Infernal aligner version 1.1 implemented in the online Ribosomal Database Project version 11.5 [6]. Alignments of protein encoding partial core gene sequences (*atpD*, *glnII*, *gyrB*, *recA* and *rpoB*) were performed as previously described [7]. Best fit substitution models were selected using ModelTest-NG [8] implemented in the CIPRES Science Gateway V.3.3 [9]. Bayesian phylogenetic analyses were performed using MrBayes version 3.2.1 with default priors [10] as described previously [11]. Maximum-likelihood (ML) phylogenetic analyses [12] were carried out using 1000 non-parametric bootstrap replications to assess support as detailed previously [7]. In all instances the topologies of trees from Bayesian and ML analyses were similar and therefore only Bayesian trees are shown in this work.

In order to reconstruct a 16S rRNA gene tree of type strains of all named species in the genus *Bradyrhizobium* (Table S1, available in the online version of this article) it was necessary to trim aligned sequence lengths to 1300 bp. The Bayesian tree of 16S rRNA gene sequences (Fig. S1) shows that all novel strains (1S1^T^, 162S2, 75S4 and 28S5; 66S1MB^T^ and 12S5) had identical sequences and were placed in a superclade represented by *B. elkanii*. It should be noted, however, that the 16S rRNA gene is highly conserved and its usefulness as a taxonomic marker for bacterial species delineation is limited [13, 14].

**Fig. 1.**
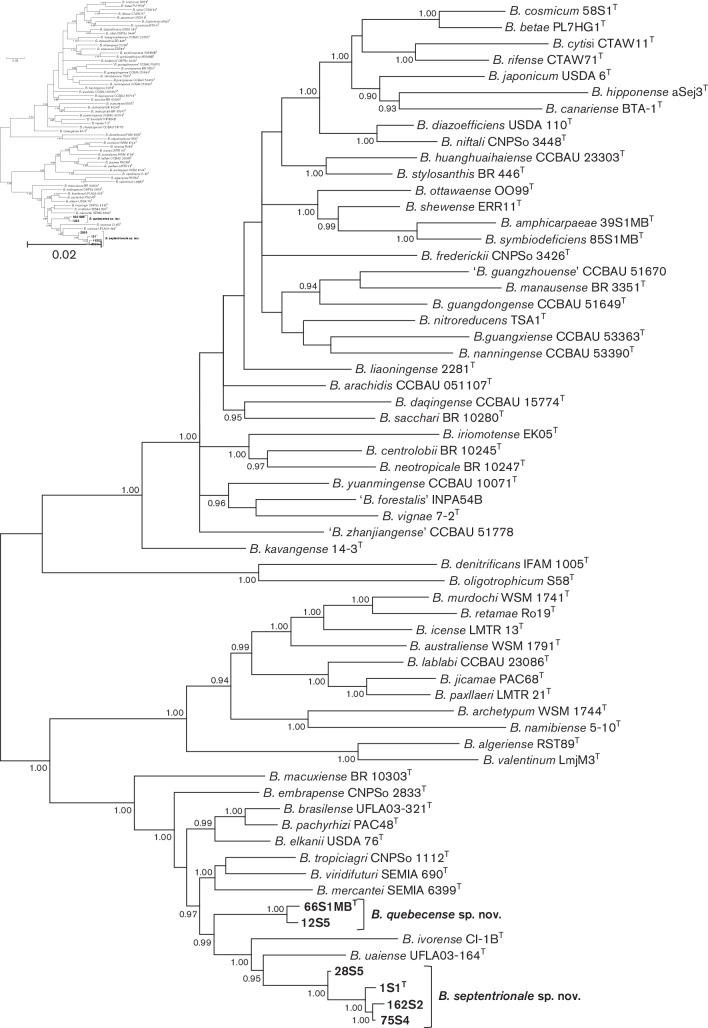
Bayesian phylogenetic tree (GTR+G+I substitution model) of *atpD–glnII–recA–gyrB–rpoB* concatenated housekeeping gene sequences for *Bradyrhizobium septentrionale* sp. nov., *Bradyrhizobium quebecense* sp. nov. and reference taxa of the genus *Bradyrhizobium*. Alignment lengths: *atpD*, 429 bp; *glnII,* 519 bp; *recA,* 417 bp; *gyrB*, 600 bp; *rpoB,* 714 bp; total, 2679 bp. Posterior probabilities ≥0.90 are shown. Bar, expected substitutions per site.

MLSA of five or more partial core gene sequences is widely used for phylogenetic analysis and delineation of species within the genus *Bradyrhizobium* [11, 14–16]. The Bayesian tree of five concatenated protein encoding core gene sequences (Fig. 1) shows that type strains representing described species in the genus *Bradyrhizobium* are grouped into four superclades represented by *B. japonicum*, *B. oligotrophicum*, *B. jicamae* and *B. elkanii* with all novel strains placed in the superclade represented by *B. elkanii*. The grouping of *Bradyrhizobium* reference strains into these four superclades is consistent with the results of other phylogenetic studies of the genus *Bradyrhizobium* [*e.g*. 1, 17, 18]. Fig. 1 further shows that the novel strains are placed in two highly supported lineages corresponding to the proposed species (*B. septentrionale* sp. nov. and *B. quebecense* sp. nov.) and each of these lineages is distinct from type strains of described *Bradyrhizobium* species.

As one or more protein encoding core gene sequences of type strains of several *Bradyrhizobium* species are not available in public databases, a supplementary phylogenetic analysis was carried out using the only two gene sequences (*recA* and *glnII*) that are available for all named species. In order to include type strains of all *Bradyrhizobium* species in the analysis, it was necessary to trim aligned sequence lengths to 411 and 519 bp for the *recA* and *glnII* genes, respectively. The Bayesian tree of concatenated *recA-glnII* gene sequences (Fig. S2) corroborates the placement of novel strains in two lineages that are distinct from named species of the genus *Bradyrhizobium*.

Data for percentage sequence similarities (16S rRNA gene sequences and five concatenated core gene sequences) of novel strains versus reference taxa, calculated by the method of Stothard [19] (Table S2) are consistent with the phylogenetic data (Figs 1 and S1).

**Fig. 2.**
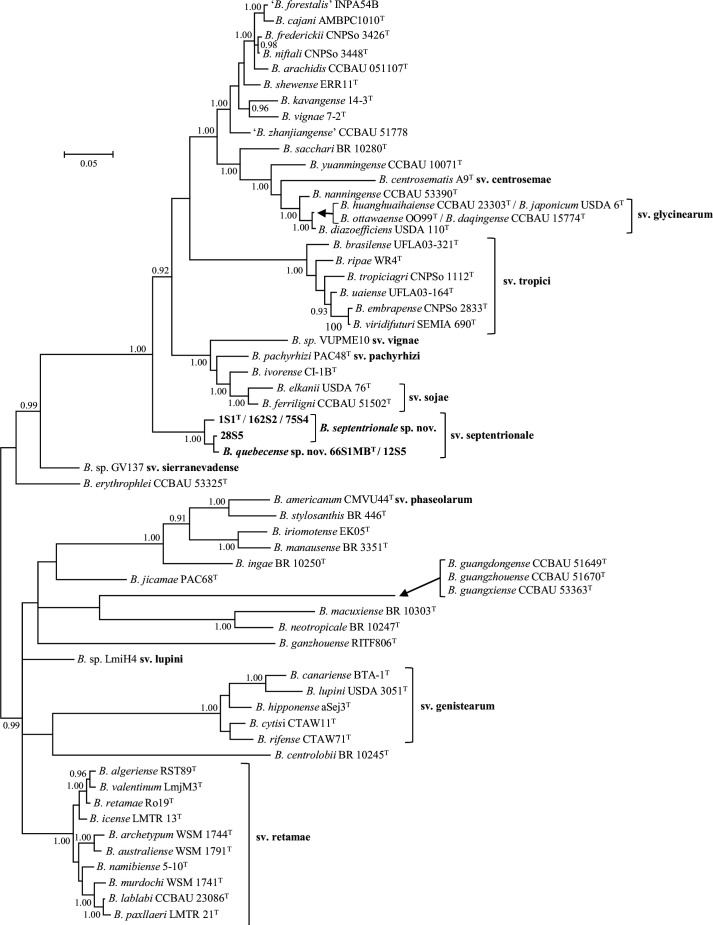
Bayesian phylogenetic tree (HKY+G substitution model) of *nodC* gene sequences (426 bp) for *Bradyrhizobium septentrionale* sp. nov. (sv. septentrionale), *Bradyrhizobium quebecense* sp. nov. (sv. septentrionale) and reference taxa of the genus *Bradyrhizobium*. Posterior probabilities ≥0.90 are shown. Bar, expected substitutions per site.

Currently twelve symbiovars have been described in the genus *Bradyrhizobium* based on distinctive nodulation (*nod*) gene sequences and symbiotic characteristics. These twelve symbiovars are shown in a Bayesian phylogenetic tree of partial sequences of the *nodC* gene for novel strains and type strains of named *Bradyrhizobium* species (Fig. 2). Consistent with our earlier work [3] the *nodC* tree shows that all novel strains are placed in a distinct and highly supported lineage corresponding to symbiovar septentrionale. A Bayesian tree of partial sequences of the nitrogen fixation (*nifH*) gene (Fig. S3) exhibits a similar topology to the *nodC* gene tree (Fig. 2) with the placement of the novel strains in a lineage that is distinct from type strains of *Bradyrhizobium* species.

## Genomics analyses

The genomes of novel strains 1S1^T^ and 162S2 (*Bradyrhizobium septentrionale* sp. nov.) and strain 66S1MB^T^ (*Bradyrhizobium quebecense* sp. nov.) were sequenced at the Genome Quebec Innovation Centre, Montreal, Canada, using the Pacific Biosciences (PacBio) Sequel single molecule real-time (SMRT) platform [20].

Table 1 shows genomic characteristics of novel and reference strains. Estimated genome coverage for novel strains was as follows: strain 1S1^T^: 93-fold with 104918 polymerase reads and an average read length of 11635 bp; strain 162S2 : 97-fold with 116227 polymerase reads and an average read length of 8985 bp; and, strain 66S1MB^T^: 125-fold with 110353 polymerase reads and an average read length of 10294 bp. The genomes of strains 1S1^T^, 162S2 and 66S1MB^T^ have sizes of 10598256, 10733150 and 9032145 bp with DNA G+C contents of 63.5, 63.4 and 63.8 mol%, respectively. As the genome assemblies of the novel strains were drafts with between two and four contigs (see Table 1) we estimated the number of plasmids in each strain based on the number of copies of *repABC* genes encoding proteins involved in plasmid replication and segregation [2, 21]. By this method, the maximum number of plasmids in novel strains was estimated to be between one and three. Further genome analyses (Table 1) using software implemented in ISfinder [22] and ISsaga [23] web-based platform indicated that abundant insertion sequences (IS) are present in the genomes of the three novel strains similar to that reported for plasmid containing and highly reiterated sequence possessing (HRS) strain, *Bradyrhizobium* sp. NK6, isolated from a soybean root-nodule in Japan [4]. Since mobile genetic elements such as plasmids are considered to be major vectors of IS transmission and IS density is often significantly higher in symbiosis islands (i.e. genomic islands containing symbiosis genes) than in bacterial chromosomes [4, 5, 24, 25], we compared the predicted IS numbers in the genomes of our novel strains with reference strains that were with and without plasmids and symbiosis islands. The data in Table 1 show an apparent relationship between predicted IS numbers and the presence of symbiosis islands and plasmids in the genomes of tested strains: high numbers of ISs (range 384–622) were predicted in novel strains 1S1^T^, 162S2, 66S1MB^T^ and HRS reference strain *Bradyrhizobium* sp. NK6 [4] possessing symbiosis islands and one or more plasmids whereas fewer ISs (69 and 104) were detected in strains *B. japonicum* USDA6^T^ [25] and *B. diazoefficiens* USDA110^T^ [24] that possess symbiosis islands but lack plasmids. Reference strains *B. symbiodeficiens* 85S1MB^T^ [26], *B. amphicarpaeae* 39S1MB^T^ [27], and, *B. cosmicum* 58S1^T^ and S23321 [18] lacking both symbiosis islands and plasmids contained the fewest ISs (range 5–15) whereas between 41 and 134 ISs were detected in *Bradyrhizobium* sp. DOA9 [28], *Bradyrhizobium* sp. BTAi1 [29] and *B. betae* PL7HG^T^ [30, 31] each possessing a single plasmid but lacking symbiosis islands.

**Table 1.** Characteristics of genome sequences of *Bradyrhizobium septentrionale* sp. nov., 1S1^T^ and 162S2, *Bradyrhizobium quebecense* sp. nov., 66S1MB^T^ and reference strains *Bradyrhizobium* sp. NK6, *Bradyrhizobium diazoefficiens* USDA 110^T^, *Bradyrhizobium japonicum* USDA 6^T^, *Bradyrhizobium* sp. DOA9, *Bradyrhizobium* sp. BTAi1, *Bradyrhizobium betae* PL7HG1^T^, *Bradyrhizobium cosmicum* 58S1^T^ and S23321, *Bradyrhizobium amphicarpaeae* 39S1MB^T^ and *Bradyrhizobium symbiodeficiens* 85S1MB^T^ Unless otherwise stated, data are from NCBI annotation databases. na, data not available.

Characteristic	Strain												
	1S1^T^	162S2	66S1MB^T^	NK6	USDA110^T^	USDA6^T^	DOA9	BTAi1	PL7HG^T^	58S1^T^	S23321	39S1MB^T^	85S1MB^T^
Genome assembly quality (no. contigs)	Draft (2)*	Draft (4)*	Draft (4)*	Complete	Complete	Complete	Draft (6)	Complete	Complete	Complete	Complete	Complete	Complete
Genome size	10598256	10733150	9032145	10475157	9105828	9207384	7850549	8493513	7419402	7304136	7231841	7044517	7039503
Symbiosis island/s	Yes	Yes	Yes	Yes	Yes	Yes	No	No	No	No	No	No	No
Plasmids (*repABC* copies)**†**	(2)	(3)	(1)	4	0	0	1‡	1	1	0	0	0	0
Predicted insertion sequences**§**	622	552	384	560||	104||	69||	134	88	41	6	5	15	7
G+C content	63.5	63.4	63.8	61.1	64.1	63.7	64.4¶	64.8	64.8	64.3	64.3	64.7	64.3
Genes (total)	10008	10125	8490	10036	8506	8681	7273¶	7673	7107	6930	6854	6576	6603
tRNAs	63	63	50	56	53	56	50¶	52	47	48	47	50	52
Pseudogenes (total)	711	726	434	1327	325	357	na	141	233	69	39	38	95

*Contigs size (bp): strain 1S1^T^: 9800512 and 797744; strain 162S2: 9880962, 440548, 332795 and 78845; strain 66S1MB^T^: 8740813, 200394, 50146 and 40792.

†Values in parentheses represent the estimated maximum number of plasmids based on *repABC* gene copies.

‡Plasmid contains symbiosis genes [28].

§Predicted IS numbers based on data from ISfinder [22] and ISsaga [23] web based platform.

||Data from Iida *et al.* [4].

¶Data from Okazaki *et al.* [28].

IS transposition and the related activities of deletion and rearrangement may contribute to pseudogenization in the bacterial host genome [5]. In this connection it is of interest to note that the data in Table 1 also show an apparent relationship between predicted IS numbers and total pseudogenes in the genomes of novel and reference strains.

**Fig. 3.**
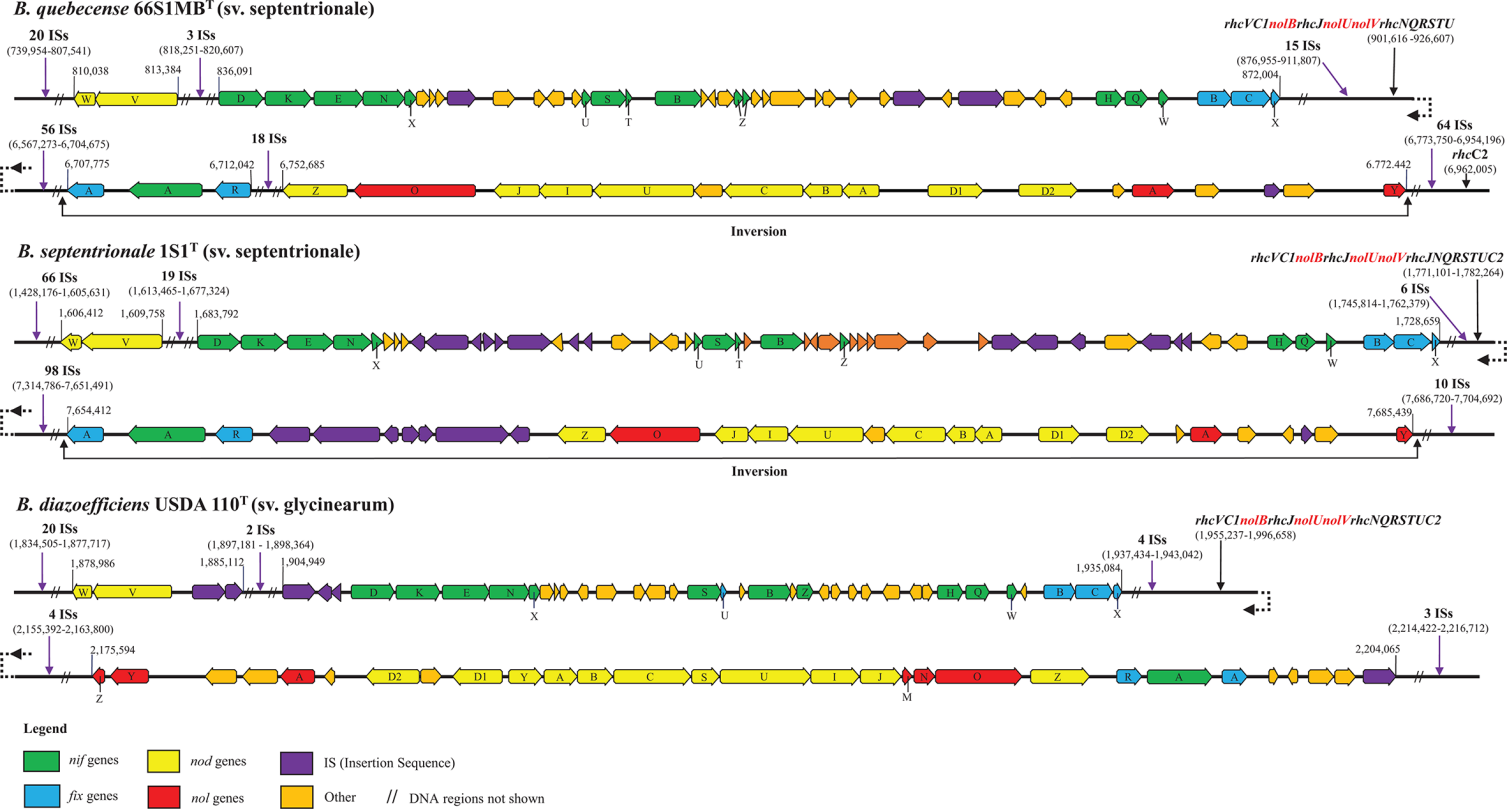
Comparative organization of nodulation (*nod*, *nol*) and nitrogen fixation (*nif*, *fix*) gene clusters of *Bradyrhizobium septentrionale* sp. nov. (sv. septentrionale) 1S1^T^, *Bradyrhizobium quebecense* sp. nov. (sv. septentrionale) 66S1MB^T^ and *Bradyrhizobium diazoefficiens* USDA110^T^ (sv. glycinearum).

Analyses were carried out using Geneious Prime version 2020.1.1 software to compare the organization of the nodulation (*nod* and *nol*) and nitrogen fixation (*nif* and *fix*) genes in the genome sequences of novel strains 1S1^T^ (sv. septentrionale) and 66S1MB^T^ (sv. septentrionale) with the corresponding genes in the symbiosis island region of reference strain *B. diazoefficiens* USDA110^T^ (sv. glycinearum) [24]. The results (Fig. 3) show an identical organization of key *nod* and *nif* genes in the genomes of the two novel strains assigned to symbiovar septentrionale. However, the *nodY* and *nolMNZ* genes present in the genome sequence of *strain* USDA 110^T^ were not detected in the genomes of novel strains 1S1^T^ and 66S1MB^T^, an observation that is consistent with a report by Passaglia [32] indicating that several strains of *B. elkanii* lack *nodY* and *nolMN* genes. Moreover, the *nod* gene cluster in novel strains 1S1^T^ and 66S1MB^T^ has undergone rearrangement and is inverted relative to the corresponding genome sequence of reference strain USDA110^T^. The entire symbiosis gene region in both novel strains has also been subjected to apparent fragmentation with the inverted gene cluster located more than 5.8 Mbp distant from the *nif-fix* gene cluster. This contrasts with reference strain USDA110^T^ where key *nod* and *nif* genes are located in a single contiguous symbiosis island (symbiosis island A) [4, 24]. Iida *et al.* [4] reported that ISs may mediate shuffling of the symbiosis island region in the HRS strain *Bradyrhizobium* sp. NK6. In this connection it is noteworthy that ISs were more abundant in the genomes of strains 1S1^T^ and 66S1MB^T^ than strain USDA110^T^ (Table 1, Fig. 3). These ISs were found to be inserted in intergenic spaces, a configuration that might be expected to be less disruptive for gene functioning than intragenic placement of ISs [33].

Type III secretion system genes (*rhc C1C2 JNQRSTUV*) required for symbiotic interaction with legume plants [34] were detected in the symbiosis gene region of both novel strains and reference strain USDA110^T^ (Fig. 3).

Average nucleotide identity (ANI) is recommended to replace DNA–DNA hybridization methods as a genome relatedness index for bacterial species delineation [13, 14, 35, 36]. The MUMmer (ANIm) algorithm implemented in the J-species web server version 3.0.20 [37] was used to estimate ANI values for genome sequences of novel strains 1S1^T^, 162S2 and 66S1MB^T^ in pair wise comparisons with the genome sequences of type strains of *Bradyrhizobium* species identified as the ten closest relatives based on the phylogenetic analyses reported in this work. The data in Table 2 shows that ANI values in comparisons with strains 1S1^T^ and 162S2 (*B. septentrionale* sp. nov.) were at or below 91.4%, and, in comparisons with strain 66S1MB^T^ (*B. quebecense* sp. nov.) were 92.3% or less. These ANI values are well below the threshold value of ~95% proposed for bacterial species circumscription [13, 35]. In contrast, the ANI value of 99.8% for the comparison of strains 1S1^T^ versus 162S2 is consistent with these strains belonging to the same species.

**Table 2.** Average Nucleotide Identity (ANI) values for pair-wise comparisons of genome sequences of *Bradyrhizobium quebecense* sp. nov. 66S1MB^T^ and *Bradyrhizobium septentrionale* 1S1^T^ and 162S2 versus closest relatives

Type strain (accession no.)	ANI (%)		
	**66S1MB^T^**	**1S1^T^**	**162S2**
***Bradyrhizobium quebecense* 66S1MB^T^ (JABWSX000000000)**	–	**91.4**	**91.3**
***Bradyrhizobium septentrionale* 1S1^T^ (JAAOLE000000000)**	**91.4**	–	**99.8**
***Bradyrhizobium septentrionale* 162S2** (**JABXFA000000000)**	**91.3**	**99.8**	–
*Bradyrhizobium embrapense* CNPSo 2833^T^ (LFIP00000000)	92.3	90.3	90.3
*Bradyrhizobium tropiciagri* CNPSo 1112^T^ (LFLZ00000000)	91.3	90.3	90.4
*Bradyrhizobium uaiense* UFLA03-164^T^ (VKHP00000000)	91.1	90.4	90.4
*Bradyrhizobium brasilense* UFLA03-321^T^ (MPVQ00000000)	91.1	90.7	90.7
*Bradyrhizobium mercantei* SEMIA 6399^T^ (MKFI00000000)	91.1	90.5	90.5
*Bradyrhizobium elkanii* USDA 76^T^ (ARAG00000000)	91.1	90.7	90.7
*Bradyrhizobium viridifuturi* SEMIA 690^T^ (LGTB00000000)	91.0	90.2	90.2
*Bradyrhizobium pachyrhizi* PAC48^T^ (LFIQ00000000)	90,9	90.6	90.6
*Bradyrhizobium ivorense* CI-1B^T^ (CAADFC00000000)	88.4	88.6	88.6
*Bradyrhizobium macuxiense* BR 10303^T^ (LNCU00000000)	88.0	88.0	88.0

**Fig. 4.**
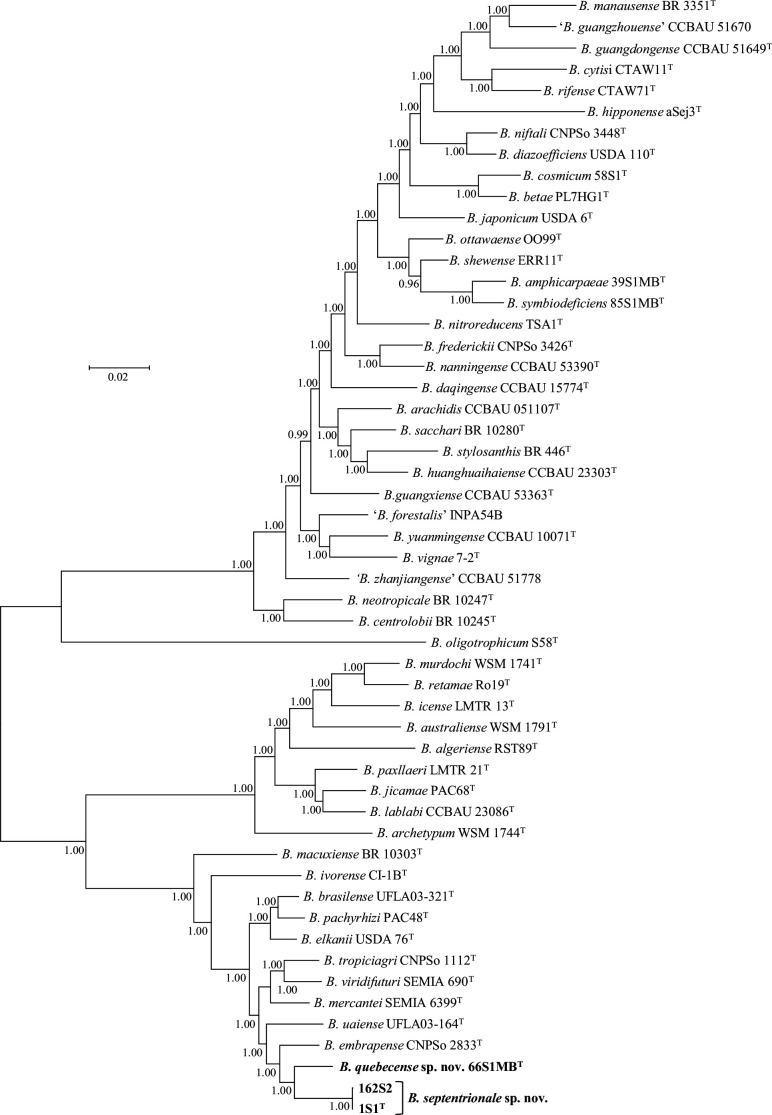
Bayesian phylogenetic tree (GTR+G+I substitution model) inferred from 52 concatenated ribosome protein subunit (*rps*) gene sequences for strains of *Bradyrhizobium septentrionale* sp. nov., *Bradyrhizobium quebecense* sp. nov. and reference taxa. Alignment length, 22703 bp. Posterior probabilities ≥0.90 are shown. Bar, expected substitutions per site.

Phylogenomic relationships were investigated using multiple gene sequences that encode bacterial ribosome protein subunits (*rps* genes) [38]. The Genome Comparator tool in the bacterial domain genome database of the BIGSdb software platform [39] was used to retrieve aligned concatenated sequences of 53 *rps* genes from the genomes of the three novel strains (1S1^T^ and 162S2, and, 66S1MB^T^) and from the type strains of 49 *Bradyrhizobium* species. For several *Bradyrhizobium* type strains, the *rpsU* locus was found to be paralogous (i.e. two or more alleles were present) and therefore this locus was excluded from the analysis. A best-fit substitution model was selected using ModelTest-NG [8]. A robust phylogenetic tree of 52 concatenated *rps* gene sequences (Fig. 4) corroborates our finding that the novel strains are placed in two highly supported lineages distinct from type strains of named *Bradyrhizobium* species. These two lineages correspond to the proposed species, *B. septentrionale* sp. nov. and *B. quebecense* sp. nov. The arrangement of taxa in Fig. 4 also confirms the placement of novel strains 1S1^T^, 162S2 and 66S1MB^T^ in a superclade represented by *B. elkanii* USDA 76^T^.

## Phenotypic characterization

Novel strains 1S1^T^ and 66S1MB^T^ produce colonies that are circular, convex, beige and ~0.1–0.25 mm diameter after 7 days growth at 28 °C on yeast extract-mannitol (YEM) agar medium [7]. Bacterial cells are Gram-stain-negative based on the KOH method of Buck [40]. Produce an alkaline reaction on YEM agar after 21 days growth at 28 °C (Table S3) which is typical of the genus *Bradyrhizobium*. Cell morphology was investigated using a transmission electron microscope (H-700, Hitachii) as described previously [11]. The results indicate that bacterial cells are rod-shaped with sub-polar flagella (Fig. S4) consistent with characteristics of the genus *Bradyrhizobium* [41].

Analysis of fatty acids was done using the Sherlock Microbial Identification System (midi) version 6.0 and the rtsba6 database as described by Yu *et al.* [11]. Data for the fatty acid profiles of novel strains 1S1^T^, 162S2, 75S4, 28S5, 66S1MB^T^, 12S5 and reference taxa are shown in Table S4. Fatty acids C_16:0_, C_18:1_ ω6c/C_18:1_ ω7c (summed feature 8), C_16:1_ ω5c, C_18:0_ and C_19:0_ cyclo ω8c were common to all novel strains, whereas C_18:0_ 10-methyl TBSA and C_19:1_ ω6c/ω7c/19cy (summed feature 7) were detected only in novel strain 1S1^T^. The predominance of fatty acids C_16:0_ and C_18:1_ ω6c/C_18:1_ ω7c (summed feature 8) in all novel strains is a characteristic feature of the genus *Bradyrhizobium* [42].

Multiple phenotypic tests including carbon source utilization and chemical sensitivity assays were carried out using BIOLOG GEN III MicroPlates (Biolog, USA) according to manufacturer’s instructions. The results (Table S3) show that strains 1S1^T^ and 66S1MB^T^ can be differentiated from one another as well as from type strains of *B. brasilense, B. elkanii, B. embrapense, B. ferriligni, B. ivorense, B. mercantei, B. pachyrhizi, B. tropiciagri, B. uaiense and B. viridifuturi* on the basis of several of these phenotypic tests.

Plant tests using Leonard jars (three replicate jars, two plants per jar) were carried out as described previously [3] using *B. diazoefficiens* USDA110^T^ as reference strain. Tests done in this study and in the previous study [3] showed that novel strains 1S1^T^, 162S2, 75S4, 28S5, 66S1MB^T^ and 12S5 (sv. septentrionale) were able to elicit nodules on the roots of soybean cultivar Glengarry but were ineffective with regard to symbiotic nitrogen fixation. Results of further tests showed that strains 1S1^T^ and 66S1MB^T^ were capable of eliciting nodules on roots of legumes native to east Canada (*Amphicarpaea bracteata*, and *Desmodium glutinosum*) but were ineffective for symbiotic nitrogen fixation. In contrast novel strain 1S1^T^ was found to nodulate and exhibit a partially effective nitrogen fixing association with the Canadian native legume, *Desmodium canadense* [3].

## Description of *Bradyrhizobium septentrionale* sp. nov. (sv. septentrionale)

*Bradyrhizobium septentrionale* (sep.ten.tri.o.na'le. L. neut. adj. *septentrionale* of the north, northern). Cells are Gram-stain-negative, aerobic, non-spore-forming rods (approx. 0.80×2.0 µm). Colonies on YEM agar medium are circular, convex, beige, translucent and ~0.1–0.25 mm in diameter after 7 days at 28 °C. Growth occurs at pH 5 but not at pH 10 (optimum ~pH 7.0). Produces an alkaline reaction on YEM agar. No growth at 10 °C or 37 °C (optimal at ~28 °C). Does not grow in the presence of 1% (w/v) NaCl. Predominant fatty acids are C_16:0_ and C_18:1_ ω*6*c/C_18:1_ ω*7c* (summed feature 8).

The type strain utilizes 41 carbon sources including dextrin, maltose, gentiobiose, stachyose, melibiose, β-methyl-D-glucoside, d-salicin, N-acetyl-D-glucosamine, N-acetyl-β-D-mannosamine, N-acetyl-D-galactosamine, N-acetyl neuraminic acid, d-mannose, d-fructose, d-galactose, 3-methyl glucose, d-fucose, l-fucose, l-rhamnose, inosine, d-aspartic acid, l-arginine, l-aspartic acid and α-keto-butyric acid. Does not utilize 30 carbon sources including d-sorbitol, glycerol, l-alanine, l-pyroglutamic acid, mucic acid, d-saccharic acid, methyl pyruvate, citric acid, l-malic acid, bromo-succinic acid, Tween 40, γ-amino-butryric acid, β-hydroxy-d,l-butyric acid, propionic acid and formic acid.

The type strain is resistant to tetrazolium violet, tetrazolium blue, nalidixic acid, lithium chloride, potassium tellurite, aztreonam, sodium butyrate and sodium bromate. Susceptible to 1% sodium lactate, fusidic acid, d-serine, troleandomycin, rifamycin SV, minocycline, lincomycin, guanidine HCl and Niaproof 4.

Elicits root nodules (ineffective for nitrogen fixation) on *Glycine max*, *Amphicarpaea bracteata*, and *Desmodium glutinosum*; elicits nodules (partially effective for nitrogen fixation) on roots of *Desmodium canadense*. The genome of the type strain contains key nodulation, nitrogen-fixation and type III secretion system genes as well as abundant insertion sequences. The type strain, 1S1^T^ (=LMG 29930^T^=HAMBI 3676^T^) was isolated from a root nodule of a soybean plant that was inoculated with root-zone soil of *Amphicarpaea bracteata* plants growing in Quebec, Canada. The DNA G+C content of the type strain is 63.5 mol% and the genome size is 10.60 Mbp.

GenBank/EMBL/DDBJ accession numbers for the whole genome and 16S rRNA, *atpD*, *glnII*, *recA*, *gyrB*, *rpoB*, *nod*C and *nifH* gene sequences of the type strain are JAAOLE000000000 and KP768787, KP768555, KP768613, KF615049, KP768729, KP768671, KF615620 and KF615667, respectively.

## Description of *Bradyrhizobium quebecense* sp. nov. (sv. septentrionale)

*Bradyrhizobium quebecense* (que.bec.en′se. N.L. neut. adj. *quebecense* of or belonging to the province of Quebec, Canada). Cells are Gram-stain-negative, aerobic, non-spore-forming rods. The type strain produces colonies on YEM agar medium that are circular, convex, beige, translucent and ~0.25 mm diameter after 7 days at 28 °C. Growth occurs at pH 5 but not at pH 10 (optimum ~pH 7.0). Produces an alkaline reaction on YEM agar. No growth at 10 °C or 37 °C (optimal at ~28 °C). Does not grow in the presence of 1% (w/v) NaCl. Predominant fatty acids are C_16:0_ and C_18:1_ ω*6*c/C_18:1_ ω*7c* (summed feature 8).

The type strain utilizes 19 carbon sources including d-sorbitol, d-mannitol, d-arabitol, myo-inositol, glycerol, d-glucose-6-PO4, d-fructose-6-PO4, pectin, d-galacturonic acid, mucic acid, quinic acid, methyl pyruvate, Tween 40, α-hydroxybutyric acid and acetoacetic acid.

Does not utilize 53 carbon sources including dextrin, maltose, gentiobiose, stachyose, melibiose, d-salicin, d-galactose, l-fucose, l-rhamnose, d-aspartic acid, l-glutamic acid, l-pyroglutamic acid, l-lactic acid, d-malic acid and acetic acid.

The type strain is resistant to troleandomycin, rifamycin SV, minocycline, tetrazolium violet, tetrazolium blue, aztreonam and sodium butyrate. The type strain is susceptible to 1% sodium lactate, fusidic acid, d-serine, lincomycin, guanidine HCl, Niaproof 4, vancomycin, nalidixic acid, lithium chloride, potassium tellurite and sodium bromate.

Elicits root nodules (ineffective for nitrogen fixation) on *Glycine max*, *Amphicarpaea bracteata*, and *Desmodium glutinosum*. The type strain, 66S1MB^T^ (=LMG 31547^T^=HAMBI 3720^T^) was isolated from a root nodule of a soybean plant that was inoculated with root-zone soil of *Amphicarpaea bracteata* plants growing in Quebec, Canada. The genome of the type strain contains key nodulation, nitrogen-fixation and type III secretion system genes as well as abundant insertion sequences. The DNA G+C content of the type strain is 63.8 mol% and the genome size is 9.03 Mbp.

GenBank/EMBL/DDBJ accession numbers for the whole genome and 16S rRNA, *atpD*, *glnII*, *recA*, *gyrB*, *rpoB*, *nod*C and *nifH* gene sequences of the type strain are JABWSX000000000 and KP768782, KP768550, KP768608, KF615025, KP768724, KP768666, KF615618 and KF615665, respectively.

## Supplementary Data

Supplementary material 1
