# Supplementary material for: Bradyrhizobium septentrionale sp. nov. (sv. septentrionale) and Bradyrhizobium quebecense sp. nov. (sv. septentrionale) associated with legumes native to Canada possess rearranged symbiosis genes and numerous insertion sequences
Source: Int J Syst Evol Microbiol. 2021 Jun 9;71(6):004831. doi: 10.1099/ijsem.0.004831 (PMC8374602; doi:10.1099/ijsem.0.004831)
Supplement: Supplementary material 1 [file ijsem-71-4831-s001.pdf]

Supplementary Data

Bradyrhizobium septentrionale **sp. nov (sv. septentrionale) and** ***Bradyrhizobium quebecense*** **sp. nov (sv. septentrionale) associated with legumes native to Canada possess rearranged symbiosis genes and numerous insertion sequences**

Eden S. P. Bromfield*and Sylvie Cloutier

Ottawa Research and Development Centre, Agriculture and Agri-Food Canada, 960 Carling Avenue, Ottawa, Ontario K1A OC6, Canada

*Corresponding author: Eden S. P. Bromfield. Email: eden.bromfield@canada.ca

Supplementary Table S1. GenBank nucleotide accession numbers for strains of *Bradyrhizobium quebecense* sp. nov., *Bradyrhizobium septentrionale* sp. nov. and reference taxa.

**Strain**	***atpD***	***glnII***	***gyrB***	***recA***	***rpoB***	**16S rRNA**	***nifH***	***nodC***
***Bradyrhizobium quebecense* 66S1MB^{T}**	KP768550	KP768608	KP768724	KF615025	KP768666	KP768782	KF615665	KF615618
***Bradyrhizobium quebecense* 12S5**	KP768536	KP768594	KP768710	KF615584	KP768652	KP768768	KF615701	KF615654
***Bradyrhizobium septentrionale* 1S1^{T}**	KP768555	KP768613	KP768729	KF615049	KP768671	KP768787	KF615667	KF615620
***Bradyrhizobium septentrionale* 162S2**	KP768567	KP768625	KP768741	KF615372	KP768683	KP768799	KF615680	KF615633
***Bradyrhizobium septentrionale* 75S4**	KP768581	KP768639	KP768755	KF615511	KP768697	KP768813	KF615692	KF615645
***Bradyrhizobium septentrionale* 28S5**	KP768540	KP768598	KP768714	KF615598	KP768656	KP768772	KF615705	KF615658
B. algeriense RST89^{T}	PYCM01000338	PYCM01000763	PYCM01000687	PYCM01000643	PYCM01000653	PYCM01000149	FJ348678	PYCM01000201
B. americanum CMVU44^{T}	-	-	NA	-	NA	KU991833	KC247135	KC247130
B. amphicarpaeae 39S1MB^{T}	KP768547	KP768605	KF615002	KP768721	KP768663	KP768779	KF615664	NA
B. arachidis CCBAU 051107^{T}	KF962683	KF962689	KF962693	KF962707	JX437682	HM107167	KF962700	KF962705
B. archetypum WSM1744^{T}	JAAVLW010000012	MK860847	MK860868	MK863448	JAAVLW010000007	MK676065	JAAVLW010000001	JAAVLW010000010
B. australiense WSM1791^{T}	JAAVLX010000013	MK860849	MK860870	MK863450	JAAVLX010000002	MK676067	JAAVLX010000005	JAAVLX010000012
B. betae PL7HG1^{T}	FM253129	AB353733	FM253217	AB353734	FM253260	NR_029104	NA	NA
B. brasilense UFLA03-321^{T}	KF452730	MPVQ01000074	KF452827	KT793142	MPVQ01000073	MPVQ01000039	MPVQ01000047	KT793173
B. cajani AMBPC1010^{T}	NA	-	NA	-	NA	KY349447	NA	KY349444
B. canariense BTA-1^{T}	FM253135	AY386765	FM253220	AY591553	FM253263	AJ558025	EU818926	EU818926
B. centrolobii BR 10245^{T}	LUUB01000107	LUUB01000131	LUUB01000063	LUUB01000078	LUUB01000064	LUUB01000105	LUUB01000073	LUUB01000057
B. centrosematis A9^{T}	-	-	NA	-	NA	KC247115	KC247139	KC247134
B. cosmicus 58S1^{T}	KP768557	KP768615	KP768731	KF615104	KP768673	KP768789	CP041656	NA
B. cytisi CTAW11^{T}	JN186289	JN186291	JN186292	JN186293	JN186288	EU561065	GU001618	EU597844
B. daqingense CCBAU 15774^{T}	HQ231289	KF962690	KF962694	KF962708	JX437676	KJ184551	KF962701	HQ231326
B. denitrificans IFAM 1005^{T}	FM253153	HM047121	FM253239	EU665419	FM253282	NR_041827	HM047125	NA
B. diazoefficiense USDA 110^{T}	BA000040	BA000040	BA000040	BA000040	BA000040	BA000040	BA000040	BA000040
B. elkanii USDA 76^{T}	AY386758	AY599117	AM418800	AY591568	EF190188	HQ233240	AB094963	AB354631
B. embrapense CNPSo 2833^{T}	LFIP02000012	LFIP0200001	LFIP0200010	LFIP0200009	LFIP0200004	AY904773	KP234518	KP234521
B. erythrophlei CCBAU 53325^{T}	NA	-	-	KF114669	-	KF1144645	KF114598	KF114576
B. ferriligni CCBAU 51502^{T}	NA	-	-	-	-	KX683400	KJ818108	KJ818109
B. forestalis INPA54B^{T}	PGVG01000048	KF452867	PGVG01000049	PGVG01000004	PGVG01000026	PGVG01000009	PGVG00000000	PGVG01000037
B. frederickii CNPSo 3426^{T}	SPQS01000024	SPQS01000012	SPQS01000005	SPQS01000004	SPQS01000016	SPQS01000017	SPQS01000036	SPQS01000032

Strain	***atpD***	***glnII***	***gyrB***	***recA***	***rpoB***	**16S rRNA**	***nifH***	***nodC***
B. ganzhouense RITF806^T	-	-	NA	-	NA	JQ796661	JX292065	JX292035
B. guangdongense CCBAU 51649^T	CP030051	CP030051	CP030051	CP030051	CP030051	CP030051	KC509130	CP030052
B. guangxiense CCBAU 53363^T	CP022219	CP022219	CP022219	CP022219	CP022219	CP022219	KC509140	CP022220
B. guangzhouense CCBAU 51670^T	CP030053	CP030053	CP030053	CP030053	CP030053	CP030053	SPQS01000036	CP030054
B. hipponense aSej3^T	VSTH01000035	VSTH01000057	VSTH01000010	VSTH01000176	VSTH01000014	VSTH01000123	VSTH01000149	VSTH01000123
B. huanghuaihaiense CCBAU 23303^T	HQ231682	KF962691	KF962695	KF962709	JX437679	HQ231463	KF962702	KF962706
B. icense LMTR 13^T	KF896192	KF896175	KF896201	JX943615	CP016428	KF896156	KF896161	KF896159
B. ingae BR 10250^T	-	-	-	-	-	KF927043	-	KF927054
B. iriomotense EK05^T	AB300994	AB300995	HQ873308	AB300996	HQ587646	AB300992	AB300998*	AB301000
B. ivorense CI-1B^T	CAADFC020000004	CAADFC020000018	CAADFC020000001	CAADFC020000009	CAADFC020000027	CAADFC020000004	CAADFC020000008	CAADFC020000011
B. japonicum USDA 6^T	AM168320	HQ587875	AM418801	AM168341	AY242830	AB510002	HM047126	AB354632
B. jicamae PAC68^T	FJ428211	FJ428204	HQ873309	HQ587415	HQ587647	AY624134	HM047127	HQ588109
B. kavangense 14-3^T	KY753592	KM378446	KX661397	KM378399	KM378311	KP899562	-	KT033402
B. lablabi CCBAU 23086^T	GU433473	GU433498	KF962696	KF962710	JX437677	GU433448	KF962703	GU433565
B. liaoningense 2281^T	FM253137	AY386775	FM253223	AY591564	FM253266	AJ250813	EU818925	NA
B. lupini USDA 3051^T	-	-	-	-	NA	KM114861	NA	KM114864
B. macuxiense BR 10303^T	LNCU01000024	LNCU01000062	LNCU01000041	LNCU01000014	LNCU01000011	LNCU01000022	LNCU00000000	KX527945
B. manausense BR 3351^T	LJYG01000004	LJYG01000112	LJYG01000105	LJYG01000054	LJYG01000045	HQ641226	LJYG00000000	LJYG01000088
B. mercantei SEMIA 6399^T	MKFI01000006	KX690621	KX690617	KX690615	MKFI01000001	FJ025102	KX690625	NA
B. murdochi WSM 1741^T	AXAU01000008	MK860844	MK860865	MK863445	AXAU01000011	MK676062	AXAU01000021	AXAU01000032
B. namibiense 5-10^T	KX661387	KM378440	KX661393	KM378377	KM378306	KX661401	-	KX661399
B. nanningense CCBAU 53390^T	LBJC01000078	LBJC01000005	LBJC01000024	LBJC01000019	LBJC01000081	LBJC01000082	LBJC01000002	LBJC01000020
B. neotropical BR 10247^T	LSEF01000046	KJ661700	LSEF01000100	KF785992	KF983829	LSEF01000032	LSEF00000000	KJ661727
B. niftali CNPSo 3448^T	SPQT01000036	SPQT01000002	SPQT01000008	SPQT01000004	SPQT01000028	SPQT01000015	SPQT01000005	SPQT01000027
B. nitroreducens TSA1^T	LFJC01000003	LFJC01000003	LFJC01000003	LFJC01000003	LFJC01000003	LFJC01000003	AB542336	NA
B. oligotrophicum S58^T	JQ619232	JQ619233	KF962697	JQ619231	KF962713	JQ619230	AP012603	NA
B. ottawaense OO99^T	HQ455212	HQ587750	HQ873179	HQ587287	HQ587518	JN186270	JN186287	HQ587980
B. pachyrhizi PAC48^T	FJ428208	FJ428201	HQ873310	HQ587416	HQ587648	AY624135	HM047124	HQ588110
B. paxllaeri LMTR 21^T	CP042968	CP042968	CP042968	CP042968	CP042968	AY923031	DQ085619	CP042968
B. retamae Ro19 ^{T}	KC247101	KC247108	KF962698	KF962711	KF962714	LLYA01000002	KF962704	KC247112

Strain	*atpD*	*glnII*	*gyrB*	*recA*	*rpoB*	16S rRNA	*nifH*	*nodC*
B. rifense CTAW71[T]	GU001617	KF962692	KF962699	KF962712	KF962715	EU561074	GU001627	EU597853
B. ripae WR4[T]	NA	-	-	-	-	MF593082	NA	MF593106
B. sacchari BR 10280[T]	KX065107	KX065099	LWIG01000001	KX065095	LWIG01000014	KF113091	LWIG00000000	KF196792
B. shewense ERR11[T]	FMAI01000019	FMAI01000022	FMAI01000013	FMAI01000022	FMAI01000007	FMAI01000022	FMAI00000000	FMAI01000039
B. stylosanthis BR 446[T]	LVEM01000002	LVEM01000001	LVEM01000002	LVEM01000001	LVEM01000001	LVEM01000016	KU724157	KU724160
B. subterraneum 58 2-1[T]	-	-	-	-	-	KP308152	-	NA
B. symbiodeficiens 85S1MB[T]	KP768551	KP768609	KP768725	KF615036	KP768667	KP768783	NA	NA
B. tropiciagri CNPSo 1112[T]	LFLZ01000050	LFLZ01000067	LFLZ01000066	LFLZ01000039	LFLZ01000008	LFLZ01000084	HQ259540	KP234520
B. uaiense UFLA03-164[T]	VKHP01000008	VKHP01000010	VKHP01000007	VKHP01000115	VKHP01000007	VKHP01000252	VKHP01000057	VKHP01000001
B. valentinum LmjM3[T]	LLXX01000203	LLXX01000118	LLXX01000044	LLXX01000017	LLXX01000029	JX514883	KF806461	LLXX01000153
B. vignae 7-2[T]	RDQF01000079	RDQF01000005	RDQF01000001	RDQF01000045	RDQF01000055	RDQF01000051	RDQF01000014	RDQF01000031
B. viridifuturi SEMIA 690[T]	LGTB01000039	LGTB01000012	LGTB01000021	LGTB01000025	LGTB01000001	FJ025107	KR149137	LGTB01000005
B. yuanmingense CCBAU 10071[T]	FM253140	AY386780	FM253226	AM168343	FM253269	AB509380	EU818927	AB354633
B. zhanjiangense CCBAU 51778[T]	CP022221	CP022221	CP022221	CP022221	CP022221	CP022221	RDQF01000014	CP022221
B. sp. LmiH4 sv. lupini	NA	NA	NA	NA	NA	NA	NA	KX272805
B. sp. sv. sierranevadense GV137	NA	NA	NA	NA	NA	NA	NA	KF483556
B. sp. sv. vignae VUPME10	NA	NA	NA	NA	NA	NA	NA	HG940520

NA, Not applicable, or, sequence not available in public databases.

\- Sequence not used in phylogenetic analyses either because sequence too short, or, one or more gene sequences for the MLSA of five housekeeping genes not available in databases.

Supplementary Table S2. Sequence similarities (%) for 16S rRNA genes and five concatenated housekeeping genes of *Bradyrhizobium quebecense* sp. nov. 66S1MB^T (1), *Bradyrhizobium septentrionale* sp. nov. 1S1^T (2) and 162S2 (3) versus six novel strains and type strains of closest relatives. NA, sequence not available in public databases.

**Strain**	**Sequence similarity (%) (coverage if not 100%*)**					
	**16S rRNA (1422 bp)**			**Five genes† (2679 bp)**		
	**1**	**2**	**3**	**1**	**2**	**3**
***Bradyrhizobium quebecense* 66S1MB^T**	-	100‡	100‡	-	96.7	96.9
***Bradyrhizobium quebecense* 12S5**	99.9‡ (95)	99.9‡ (95)	99.9‡ (95)	99.5	96.8	96.9
***Bradyrhizobium septentrionale* 1S1^T**	100‡	-	100‡	96.7	-	99.4
***Bradyrhizobium septentrionale* 162S2**	100‡	100‡	-	96.9	99.4	-
***Bradyrhizobium septentrionale* 75S4**	100‡ (95)	100‡ (95)	100‡ (95)	97.0	99.6	99.7
***Bradyrhizobium septentrionale* 28S5**	100‡ (95)	100‡ (95)	100‡ (95)	96.0	98.9	98.7
Bradyrhizobium brasilense UFLA 03-321^T	99.5‡	99.5‡	99.5‡	96.4	95.9	95.7
Bradyrhizobium elkanii USDA 76^T	99.5	99.5	99.5	96.0	96.2	96.0
Bradyrhizobium embrapense CNPSo 2833^T	99.8	99.8	99.8	96.5	96.1	95.9
Bradyrhizobium erythrophlei CCBAU 53325^T	99.8 (95)	99.8 (95)	99.8 (95)	NA	NA	NA
Bradyrhizobium ferriligni CCBAU 51502^T	99.4‡ (93)	99.4‡ (93)	99.4‡ (93)	NA	NA	NA
Bradyrhizobium ivorense CI-1B^T	99.2	99.2	99.2	94.9	94.6	94.4
Bradyrhizobium macuxiense BR 10303^T	99.3‡	99.3‡	99.3‡	94.7	94.7	94.6
Bradyrhizobium mercantei SEMIA 6399^T	99.8	99.8	99.8	95.9	95.9	95.6
Bradyrhizobium pachyrhizi PAC48^T	99.5	99.5	99.5	96.5	96.1	95.9
Bradyrhizobium ripae WR4^T	99.5	99.5	99.5	NA	NA	NA
Bradyrhizobium tropiciagri CNPSo 1112^T	99.5	99.5	99.5	96.4	96.2	96.1
Bradyrhizobium uaiense UFLA03-164^T	99.9‡	99.9‡	99.9‡	96.0	95.8	95.6
Bradyrhizobium viridifuturi SEMIA 690^T	99.8	99.8	99.8	96.2	95.9	95.7

* Short sequences.

† Five concatenated protein encoding core genes: *atpD-glnII-gyrB-recA-rpoB*

‡ 16S rRNA gene similarities calculated using sequences from GenBank as reference sequences not available in EzBioCloud database [Yoon et al. *Int J Syst Evol Microbiol* 2017; 67:1613–1617].

Supplementary Table S3. Phenotypic characteristics of **1**. *B.septentrionale* sp. nov. 1S1^{T}, **2.** *B. quebecense* 66S1MB^{T}, **3**. *B. brasilense* UFLA03-321^{T}, **4**. *B. elkanii* USDA 76^{T}, **5**. *B. embrapense* CNPSo 2833^{T}, **6**. *B. ferriligni* CCBAU 51502^{T}, **7**. *B. ivorense* CI-1B^{T}, **8**. *B. mercantei* SEMIA 6399^{T}, **9**. *B. pachyrhizi* PAC48^{T}, **10**. *B. tropiciagri* CNPSo 1112^{T}, **11**. *B. uaiense* UFLA03-164^{T} and **12**. *B. viridifuturi* SEMIA 690^{T}. Values are based on three replicates. ND, not determined.

**Characteristic**	**1**	**2**	**3**	**4**	**5**	**6**	**7**	**8**	**9**	**10**	**11**	**12**	**Characteristic**	**1**	**2**	**3**	**4**	**5**	**6**	**7**	**8**	**9**	**10**	**11**	**12**
C-source utilization (BIOLOG)[a]																									
Dextrin	+	-	-	-	-	-	-	-	-	-	-	-	Glycyl-L-Proline	-	-	-	-	-	-	-	+	-	-	-	-
D-Maltose	±	-	-	-	-	-	-	-	±	-	-	-	L-Alanine	-	-	-	-	±	-	-	-	-	-	-	-
D-Trehalose	-	-	-	-	-	-	-	-	-	-	-	-	L-Arginine	+	-	-	-	-	-	-	-	-	-	-	-
D-Cellobiose	-	-	-	-	-	-	-	-	-	-	-	-	L-Aspartic Acid	±	-	-	-	-	-	-	-	-	-	-	-
Gentiobiose	+	-	-	-	-	-	-	-	-	-	-	-	L-Glutamic Acid	±	-	-	-	±	-	-	-	±	-	-	-
Sucrose	-	-	-	-	-	-	-	-	-	-	-	-	L-Histidine	-	-	-	-	-	-	-	-	-	-	-	-
D-Turanose	-	-	-	-	-	-	-	-	-	-	-	-	L-Pyroglutamic Acid	-	-	-	+	+	-	+	+	+	-	-	+
Stachyose	±	-	-	-	-	-	-	-	-	-	-	-	L-Serine	-	-	-	-	-	-	-	-	-	-	-	-
D-Raffinose	-	-	-	-	-	-	-	-	-	-	-	-	Pectin	±	+	±	+	-	-	±	+	±	±	-	±
α-D-Lactose	-	-	-	-	-	-	-	-	-	-	-	-	D-Galacturonic Acid	+	+	+	+	±	+	-	+	+	+	+	±
D-Melibiose	+	-	-	-	-	-	-	-	-	-	-	-	L-Galactonic Acid Lactone	+	+	+	+	±	±	+	+	+	+	+	±
β-Methyl-DGlucoside	+	-	-	-	-	-	-	-	-	-	-	-	D-Gluconic Acid	+	+	+	+	+	-	+	+	+	-	-	+
D-Salicin	+	-	-	-	-	-	-	-	-	-	-	-	D-Glucuronic Acid	+	+	+	±	±	±	-	±	±	+	±	-
N-Acetyl-DGlucosamine	+	-	-	-	-	-	-	-	-	-	-	-	Glucuronamide	+	+	+	+	+	+	+	+	+	+	+	+
N-Acetyl-β-DMannosamine	+	-	-	-	-	-	-	-	-	-	-	-	Mucic Acid	-	+	+	+	+	-	±	+	+	-	+	+
N-Acetyl-DGalactosamine	+	-	-	-	-	-	-	-	-	-	-	-	Quinic Acid	+	±	±	±	±	-	+	+	±	-	-	-
N-Acetyl Neuraminic Acid	±	-	-	-	-	-	-	-	-	-	-	-	D-Saccharic Acid	-	-	-	+	+	-	±	+	+	-	±	+
α-D-Glucose	-	-	-	-	-	-	-	-	-	-	-	-	p-HydroxyPhenylacetic Acid	±	-	-	-	±	-	-	-	±	-	-	-
D-Mannose	+	-	-	-	-	-	-	-	-	-	-	-	Methyl Pyruvate	-	+	+	+	+	±	+	+	+	±	+	+
D-Fructose	+	-	-	-	-	-	-	-	-	-	-	-	D-Lactic Acid Methyl Ester	-	-	-	-	-	-	-	-	-	-	-	-
D-Galactose	+	-	+	-	+	-	+	+	±	-	-	±	L-Lactic Acid	+	-	-	+	±	-	+	-	-	-	-	-
3-Methyl Glucose	+	-	-	-	-	-	-	-	-	-	-	-	Citric Acid	-	-	-	+	+	-	-	±	-	-	-	+
D-Fucose	+	-	+	+	+	±	+	+	+	-	-	+	α-Keto-Glutaric Acid	-	-	-	+	+	-	±	±	-	-	-	+
L-Fucose	+	-	-	-	±	-	+	-	±	-	-	±	D-Malic Acid	+	-	-	±	±	-	±	±	-	-	-	+
L-Rhamnose	+	-	-	-	-	-	-	-	±	-	-	-	L-Malic Acid	-	-	-	±	±	-	±	+	-	-	-	+
Inosine	+	-	-	-	-	-	-	-	-	-	-	-	Bromo-Succinic Acid	-	-	±	+	+	-	+	±	-	-	-	±
D-Sorbitol	-	±	+	-	+	±	+	±	±	±	-	-	Tween 40	-	+	+	+	+	±	±	+	+	±	±	+
D-Mannitol	+	+	+	+	+	+	+	+	+	+	+	-	γ-Amino-Butryric Acid	-	-	-	±	-	-	±	-	±	±	±	-
D-Arabitol	-	+	+	+	+	+	+	+	+	+	±	±	α-HydroxyButyric Acid	+	+	-	-	±	-	-	-	-	-	-	-
myo-Inositol	+	+	+	-	±	+	-	-	±	+	±	-	β-Hydroxy-D,LButyric Acid	-	-	-	+	+	-	±	+	±	±	-	+
Glycerol	-	+	+	+	+	±	+	+	+	+	+	±	α-Keto-Butyric Acid	+	-	-	-	-	-	-	-	-	-	-	-
D-Glucose- 6-PO4	+	+	+	-	±	+	±	±	+	+	-	-	Acetoacetic Acid	±	±	-	-	±	-	-	-	-	±	-	-
D-Fructose- 6-PO4	+	+	+	+	+	+	+	+	+	+	+	-	Propionic Acid	-	-	-	-	±	-	-	-	-	-	-	+
D-Aspartic Acid	±	-	-	-	-	-	-	-	±	-	-	-	Acetic Acid	±	-	-	+	±	-	±	-	-	-	-	+
D-Serine	-	-	-	-	-	-	-	-	±	-	-	-	Formic Acid	-	-	-	+	-	-	+	-	-	-	-	+
Gelatin	-	-	-	-	-	-	-	-	-	-	-	-													
Chemical Sensitivity (BIOLOG)[a]																									
1% Sodium Lactate	-	-	-	-	-	-	-	-	-	-	-	-	Vancomycin	-	-	-	±	±	±	-	±	±	-	-	+
Fusidic Acid	-	-	-	-	-	-	-	-	-	-	-	-	Tetrazolium Violet	±	+	+	+	+	+	+	+	+	+	+	+
D-Serine	-	-	-	-	-	-	-	+	-	-	-	-	Tetrazolium Blue	±	+	+	+	+	+	+	+	+	+	+	+
Troleandomycin	-	+	-	+	+	±	±	+	±	±	±	+	Nalidixic Acid	+	-	-	±	±	±	-	±	-	-	-	+
Rifamycin SV	-	+	±	+	+	+	+	+	±	+	-	+	Lithium Chloride	+	-	-	-	-	-	-	-	-	-	-	-
Minocycline	-	+	-	+	+	+	+	+	±	+	+	+	Potassium Tellurite	+	-	-	+	-	-	-	-	±	-	-	+
Lincomycin	-	-	-	±	±	-	-	-	-	-	-	+	Aztreonam	+	±	-	+	±	±	±	+	±	±	-	+
Guanidine HCl	-	-	-	-	-	-	-	-	-	-	-	-	Sodium Butyrate	+	±	-	-	±	±	±	-	±	±	-	-
Niaproof 4	-	-	-	-	-	-	-	-	-	-	-	-	Sodium Bromate	+	-	-	+	±	±	-	-	±	-	-	-
Growth on YEM agar medium:																									
10 °C	-	-	-	-	-	-	ND	-	-	-	ND	ND													
37 °C	-	-	-	-	+	-	ND	-	-	-	ND	ND													
pH 5	+	+	+	+	+	+	ND	+	+	+	ND	ND													
pH 10	-	-	+	+	+	±	ND	+	+	-	ND	ND													
1% NaCl	-	-	-	-	±	-	ND	-	-	-	ND	ND													
Acid/alkali production (pH)[b]	8.0	8.0	ND	ND	ND	ND	ND	ND	ND	ND	ND	ND													

[a] BIOLOG GEN III MicroPlates (120 hours incubation at 28 ºC); +, Positive; ±, weak; −, negative.

[b] Acid/alkali production after 21 days at 28 C; uninoculated control, pH 7.1.

Supplementary Table S4. Fatty acid profiles of *Bradyrhizobium* strains: **1.** *B. quebecense* sp. nov 66S1MB^{T}; **2.** *B. quebecense* sp. nov 12S5; **3**. *B. septentrionale* sp. nov 1S1^{T}; **4**. *B. septentrionale* sp. nov 162S2; **5**. *B. septentrionale* sp. nov 75S4; **6**. *B. septentrionale* sp. nov 28S5; **7**. *B. brasilense* UFLA03-321^{T}; **8.** *B. tropiciagri* CNPSo 1112^{T}; **9.** *B. mercantei* SEMIA 6399^{T}; **10**. *B. ferriligni* CCBAU 51502^{T}; **11.** *B. elkanii* USDA 76^{T}.

**Fatty Acid**	**Strain**										
	**1**	**2**	**3**	**4**	**5**	**6**	**7**	**8†**	**9‡**	**10‡**	**11†**
9:0		1.02									
12:0		1.00			1.32	0.94					
14:0		0.43			0.66	0.32					
15:1 ω8c			2.39								
16:0	10.48	10.16	11.24	10.71	12.45	12.32	9.94	15.83	13.41	17.70	11.07
16:1 ω5c	0.49	1.42	3.50	2.71	2.92	5.78	0.84			0.76	
17:0 cyclo	0.60	0.72			0.62	0.72	0.63				
18:0	1.72		1.93	1.35	3.24	1.90	1.31				
18:0 10-methyl, TBSA			5.58								
18:1 w5c	0.39	0.34				0.41					
18:1ω7c 11-methyl						0.41	1.75				
18:3 w6c (6,9,12)		0.52									
19:0 iso		1.30			2.43	0.68	0.54				
19:0 cyclo ω8c	2.76	6.42	6.24	2.77	5.31	8.93	38.19	15.09	13.04	30.07	13.67
20:0	0.38	0.50			0.65	0.33					
20:2 w6,9c							1.38				
Summed Feature 3*	1.20	0.73		1.53	1.14	0.92					
Summed Feature 7*			3.61				0.47				
Summed Feature 8*	81.98	72.88	65.51	80.93	69.26	66.36	44.95	69.08	72.67	50.03	75.26

* Summed Features are fatty acids that cannot be resolved reliably from another fatty acid using the chromatographic conditions chosen. The MIDI system groups these fatty acids together as one feature with a single percentage of the total. Summed Feature 3, 16:1ω6c/16:1ω7c; Summed Feature 7, 19:1 ω6c/ω7c/19cy; Summed Feature 8, 18:1ω6c/18:1ω7c.

† Data from: Delamuta *et al. Int J Syst Evol Microbiol* 2015; 65:4424-4433

‡ Data from: Helene *et al*. *Int J Syst Evol Microbiol* 2017; 67:1827-1834

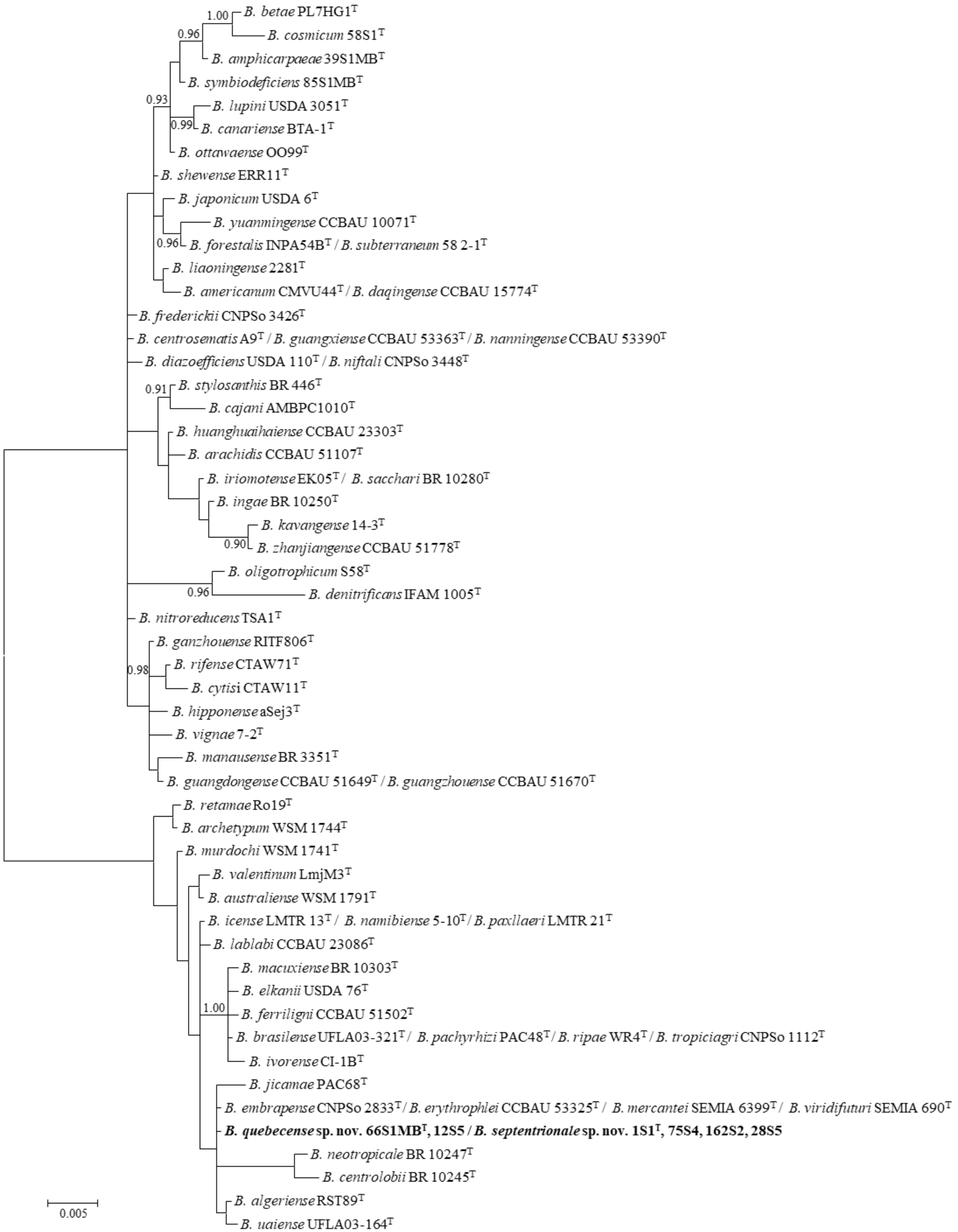
1.00
0.96
0.93
0.99
0.96
0.91
0.90
0.96
0.98
1.00
B. betae PL7HG1T
B. cosmicum 58S1T
B. amphicarpaeae 39S1MBT
B. symbiodeficiens 85S1MBT
B. lupini USDA 3051T
B. canariense BTA-1T
B. ottawaense OO99T
B. shewense ERR11T
B. japonicum USDA 6T
B. yuanmingense CCBAU 10071T
B. forestalis INPA54BT / B. subterraneum 58 2-1T
B. liaoningense 2281T
B. americanum CMVU44T / B. daqingense CCBAU 15774T
B. fredericksii CNPSo 3426T
B. centrosematis A9T / B. guangxiense CCBAU 53363T / B. nanningense CCBAU 53390T
B. diazoefficiens USDA 110T / B. niftali CNPSo 3448T
B. stylosanthis BR 446T
B. cajani AMBPC1010T
B. huanghuaihaiense CCBAU 23303T
B. arachidis CCBAU 51107T
B. iriomotense EK05T / B. sacchari BR 10280T
B. ingae BR 10250T
B. kavangense 14-3T
B. zhanjiangense CCBAU 51778T
B. oligotrophicum S58T
B. denitrificans IFAM 1005T
B. nitroreducens TSA1T
B. ganzhouense RITF806T
B. rifense CTAW71T
B. cytisi CTAW11T
B. hipponense aSej3T
B. vignae 7-2T
B. manausense BR 3351T
B. guangdongense CCBAU 51649T / B. guangzhouense CCBAU 51670T
B. retamae Ro19T
B. archetypum WSM 1744T
B. murdochi WSM 1741T
B. valentinum LmjM3T
B. australiense WSM 1791T
B. icense LMTR 13T / B. namibiense 5-10T / B. paxllaeri LMTR 21T
B. lablabi CCBAU 23086T
B. macuxiense BR 10303T
B. elkanii USDA 76T
B. ferriligni CCBAU 51502T
B. brasilense UFLA03-321T / B. pachyrhizi PAC48T / B. ripae WR4T / B. tropiciagri CNPSo 1112T
B. ivorense CI-1BT
B. jicamae PAC68T
B. embrapense CNPSo 2833T / B. erythrophlei CCBAU 53325T / B. mercantei SEMIA 6399T / B. viridifuturi SEMIA 690T
B. quebecense sp. nov. 66S1MBT, 12S5 / B. septentrionale sp. nov. 1S1T, 75S4, 162S2, 28S5
B. neotropicale BR 10247T
B. centrolobii BR 10245T
B. algeriense RST89T
B. uaiense UFLA03-164T
0.005

Supplementary Fig. S1. Bayesian phylogenetic tree of 16S rRNA gene sequences for *Bradyrhizobium septentrionale* sp. nov., *Bradyrhizobium quebecense* sp. nov. and reference taxa (HKY + G + I substitution model). Only posterior probabilities ≥ 90% are shown. Scale bar represents expected number of substitutions per site.

To include all species of *Bradyrhizobium* in the tree it was necessary to trim alignment lengths of 16S rRNA gene sequences to 1300 bp.

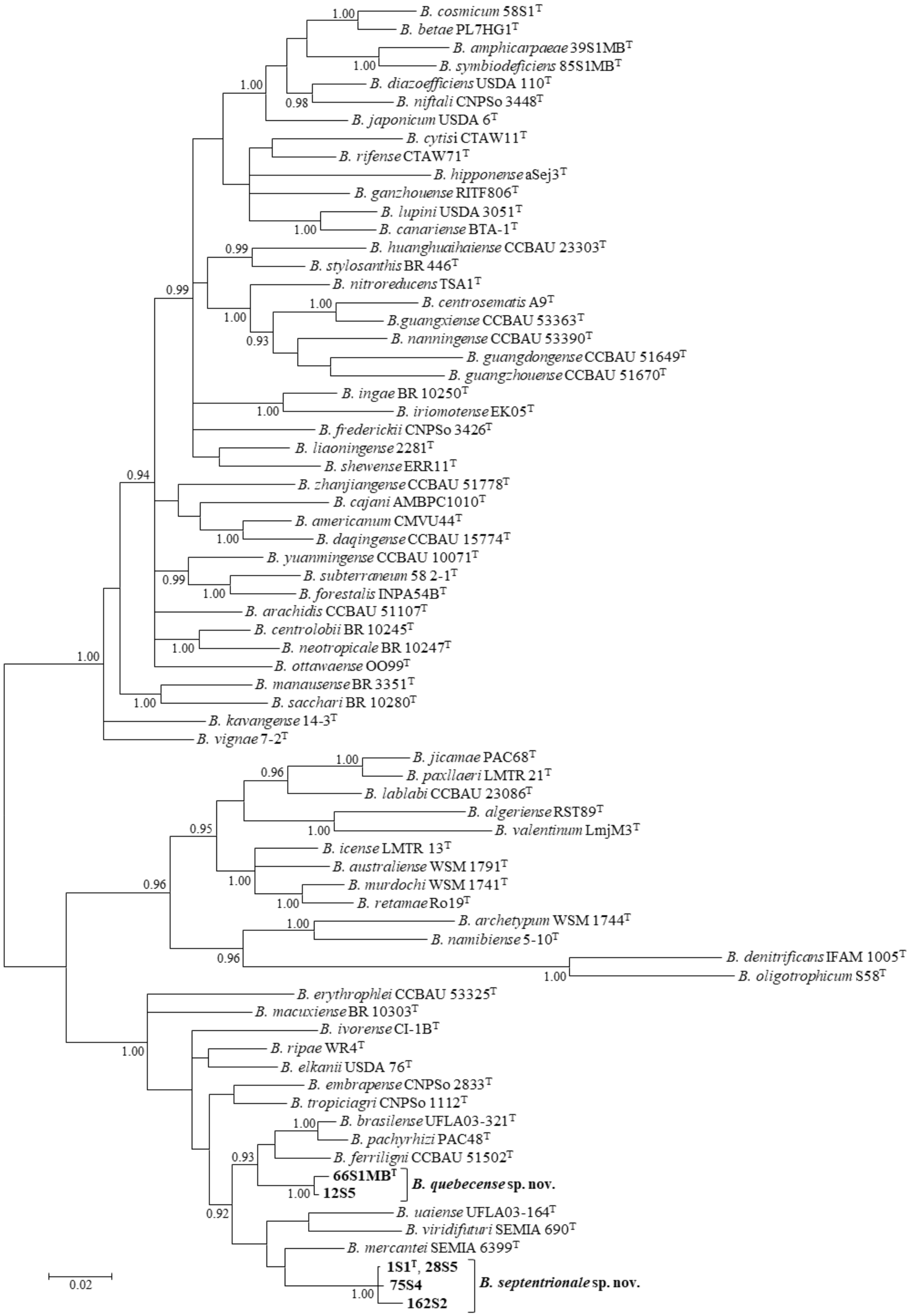

B. cosmicum 58S1T
B. betae PL7HG1T
B. amphicarpaeae 39S1MBT
B. symbiodeficiens 85S1MBT
B. diazoefficiens USDA 110T
B. niftali CNPSo 3448T
B. japonicum USDA 6T
B. cytisi CTAW11T
B. rifense CTAW71T
B. hipponense aSej3T
B. ganzhouense RITF806T
B. lupini USDA 3051T
B. canariense BTA-1T
B. huanghuaihaiense CCBAU 23303T
B. stylosanthis BR 446T
B. nitroreducens TSA1T
B. centrosematis A9T
B.guangxiense CCBAU 53363T
B. nanningense CCBAU 53390T
B. guangdongense CCBAU 51649T
B. guangzhouense CCBAU 51670T
B. ingae BR 10250T
B. iriomotense EK05T
B. frederickii CNPSo 3426T
B. liaoningense 2281T
B. shewense ERR11T
B. zhanjiangense CCBAU 51778T
B. cajani AMBPC1010T
B. americanum CMVU44T
B. daqingense CCBAU 15774T
B. yuanmingense CCBAU 10071T
B. subterraneum 58 2-1T
B. forestalis INPA54BT
B. arachidis CCBAU 51107T
B. centrolobii BR 10245T
B. neotropicale BR 10247T
B. ottawaense OO99T
B. manausense BR 3351T
B. sacchari BR 10280T
B. kavangense 14-3T
B. vignae 7-2T
B. jicamae PAC68T
B. paxllaeri LMTR 21T
B. lablabi CCBAU 23086T
B. algeriense RST89T
B. valentinum LmjM3T
B. icense LMTR 13T
B. australiense WSM 1791T
B. murdochi WSM 1741T
B. retamae Ro19T
B. archetypum WSM 1744T
B. namibiense 5-10T
B. denitrificans IFAM 1005T
B. oligotrophicum S58T
B. erythrophlei CCBAU 53325T
B. macuxiense BR 10303T
B. ivorense CI-1BT
B. ripae WR4T
B. elkanii USDA 76T
B. embrapense CNPSo 2833T
B. tropiciagri CNPSo 1112T
B. brasilense UFLA03-321T
B. pachyrhizi PAC48T
B. ferriligni CCBAU 51502T
66S1MBT
12S5
B. quebecense sp. nov.
B. uaiense UFLA03-164T
B. viridifuturi SEMIA 690T
B. mercantei SEMIA 6399T
1S1T, 28S5
75S4
162S2
B. septentrionale sp. nov.
1.00
0.98
0.99
0.93
0.94
0.96
0.95
0.92
0.02

Supplementary Fig. S2. Bayesian phylogenetic tree (GTR + G + I substitution model) of *recA-glnII* concatenated gene sequences (930 bp) for *Bradyrhizobium septentrionale* sp. nov., *Bradyrhizobium quebecense* sp. nov. and reference taxa of the genus *Bradyrhizobium*. Only posterior probabilities ≥ 90% are shown. Bar, expected substitutions per site.

To include all named species of *Bradyrhizobium* in the analysis, it was necessary to trim individual sequence alignment lengths to 411 and 519 bp for *recA* and *glnII*, respectively.

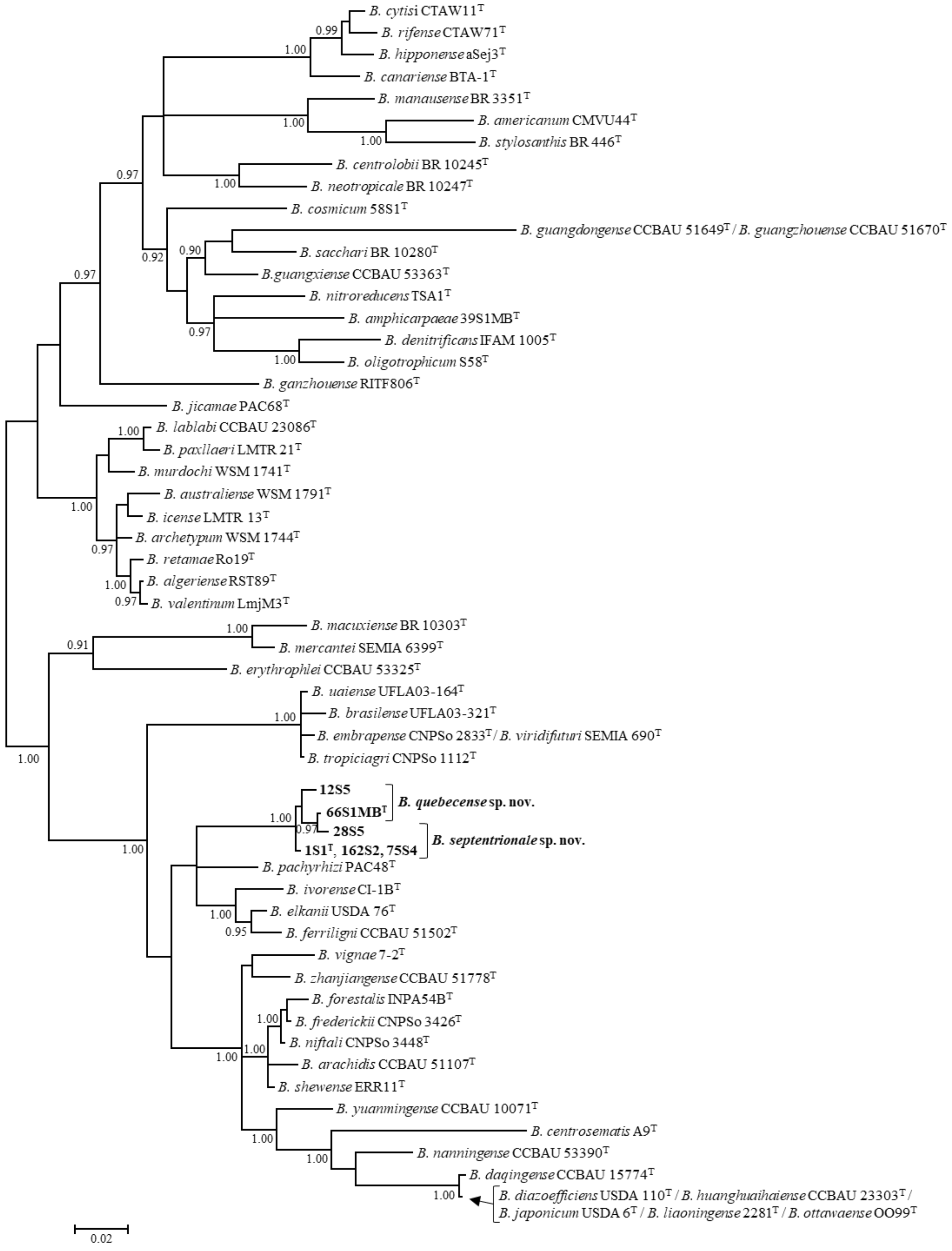
B. cytisi CTAW11^{T}
B. rifense CTAW71^{T}
B. hipponense aSej3^{T}
B. canariense BTA-1^{T}
B. manausense BR 3351^{T}
B. americanum CMVU44^{T}
B. stylosanthis BR 446^{T}
B. centrolobii BR 10245^{T}
B. neotropicale BR 10247^{T}
B. cosmicum 58S1^{T}
B. guangdongense CCBAU 51649^{T} / B. guangzhouense CCBAU 51670^{T}
B. sacchari BR 10280^{T}
B.guangxiense CCBAU 53363^{T}
B. nitroreducens TSA1^{T}
B. amphicarpaeae 39S1MB^{T}
B. denitrificans IFAM 1005^{T}
B. oligotrophicum S58^{T}
B. ganzhouense RITF806^{T}
B. jicamae PAC68^{T}
B. lablabi CCBAU 23086^{T}
B. paxllaeri LMTR 21^{T}
B. murdochi WSM 1741^{T}
B. australiense WSM 1791^{T}
B. icense LMTR 13^{T}
B. archetypum WSM 1744^{T}
B. retamae Ro19^{T}
B. algeriense RST89^{T}
B. valentinum LmjM3^{T}
B. macuxiense BR 10303^{T}
B. mercantei SEMIA 6399^{T}
B. erythrophlei CCBAU 53325^{T}
B. uaiense UFLA03-164^{T}
B. brasilense UFLA03-321^{T}
B. embrapense CNPSo 2833^{T} / B. viridifuturi SEMIA 690^{T}
B. tropiciagri CNPSo 1112^{T}
12S5
66S1MB^{T}
B. quebecense sp. nov.
28S5
1S1^{T}, 162S2, 75S4
B. septentrionale sp. nov.
B. pachyrhizi PAC48^{T}
B. ivorense CI-1B^{T}
B. elkanii USDA 76^{T}
B. ferriligni CCBAU 51502^{T}
B. vignae 7-2^{T}
B. zhanjiangense CCBAU 51778^{T}
B. forestalis INPA54B^{T}
B. frederickii CNPSo 3426^{T}
B. niftali CNPSo 3448^{T}
B. arachidis CCBAU 51107^{T}
B. shewense ERR11^{T}
B. yuanmingense CCBAU 10071^{T}
B. centrosematis A9^{T}
B. nanningense CCBAU 53390^{T}
B. daqingense CCBAU 15774^{T}
B. diazoefficiens USDA 110^{T} / B. huanghuaihaiense CCBAU 23303^{T} /
B. japonicum USDA 6^{T} / B. liaoningense 2281^{T} / B. ottawaense OO99^{T}
0.02

Supplementary Fig. S3. Bayesian phylogenetic tree (HKY+G substitution model) of *nifH* gene sequences (540 bp) for *Bradyrhizobium septentrionale* sp. nov. (sv. septentrionale), *Bradyrhizobium quebecense* sp. nov. (sv. septentrionale) and reference taxa of the genus *Bradyrhizobium.* Posterior probabilities ≥ 0.90 are shown. Bar, expected substitutions per site.

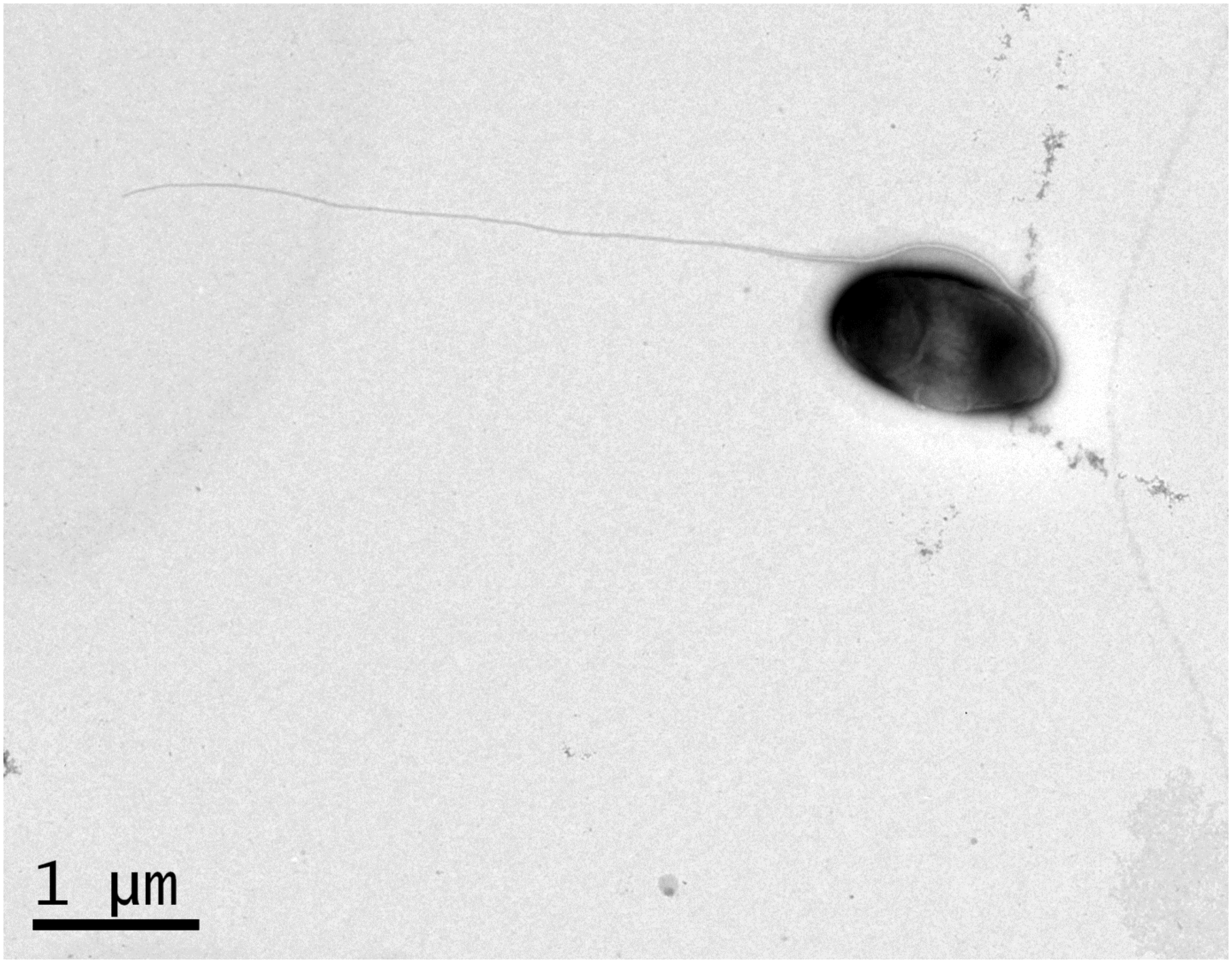

Supplementary Fig. S4. Transmission electron micrograph showing a cell of

Bradyrhizobium septentrionale sp. nov $1S1^T$ (sv. septentrionale) with sub-polar flagellum.

Average cell size, 0.8 x 2.0 µm (based on 10 measurements).
